# Exosomes as CNS Drug Delivery Tools and Their Applications

**DOI:** 10.3390/pharmaceutics14102252

**Published:** 2022-10-21

**Authors:** Ke Sun, Xue Zheng, Hongzhen Jin, Fan Yu, Wei Zhao

**Affiliations:** 1 College of Pharmacy, Nankai University, Tongyan Road, Haihe Education Park, Tianjin 300350, China; 2 College of Life Sciences, Nankai University, Weijin Road, Nankai District, Tianjin 300350, China; 3 State Key Laboratory of Medicinal Chemical Biology, Nankai University, Tongyan Road, Haihe Education Park, Tianjin 300350, China

**Keywords:** engineered exosomes, drug delivery system, CNS, blood-brain barrier

## Abstract

Central nervous system (CNS) diseases threaten the health of people all over the world. However, due to the structural and functional particularities of the brain and spinal cord, CNS-targeted drug development is rather challenging. Exosomes are small cellular vesicles with lipid bilayers that can be secreted by almost all cells and play important roles in intercellular communication. The advantages of low immunogenicity, the ability to cross the blood-brain barrier, and the flexibility of drug encapsulation make them stand out among CNS drug delivery tools. Herein, we reviewed the research on exosomes in CNS drug delivery over the past decade and outlined the impact of the drug loading mode, administration route, and engineered modification on CNS targeting. Finally, we highlighted the problems and prospects of exosomes as CNS drug delivery tools.

## 1. Introduction

The drug delivery system (DDS) can comprehensively regulate the distribution of drugs in the organism in space, time, and dose. It has multiple roles: (i) drug targeting; (ii) slow and controlled drug release; (iii) enhancing drug stability and regulating drug metabolism time; (iv) promoting drug absorption and passage through biological barriers. Modern drug delivery technology started with the advent of extended-release capsule technology in 1952, followed by oral and transdermal formulations, long-acting injectables, pegylated liposomes, drug-polymer complexes, antibody-drug conjugates, and nano-delivery systems over 70 years [1].

Delivery of drugs to the CNS has been a challenging subject to overcome. In the brain, the main biological barrier is the blood-brain barrier (BBB). Various pathways have been developed to overcome the poor penetration of the BBB [2,3,4,5,6]. However, so far, it remains a bottleneck in CNS drug development [7]. To overcome the BBB, lots of drug delivery systems have been developed, such as adeno-associated viral (AAV) vectors [8], erythrocyte membrane-encapsulated nanocarriers [9], cell-based delivery nanocarriers [10], extracellular vesicles (EVs) [11], injectable hydrogels [12], and immunomodulators [13]. Among them, EVs performed surprisingly well.

**Figure 1:**
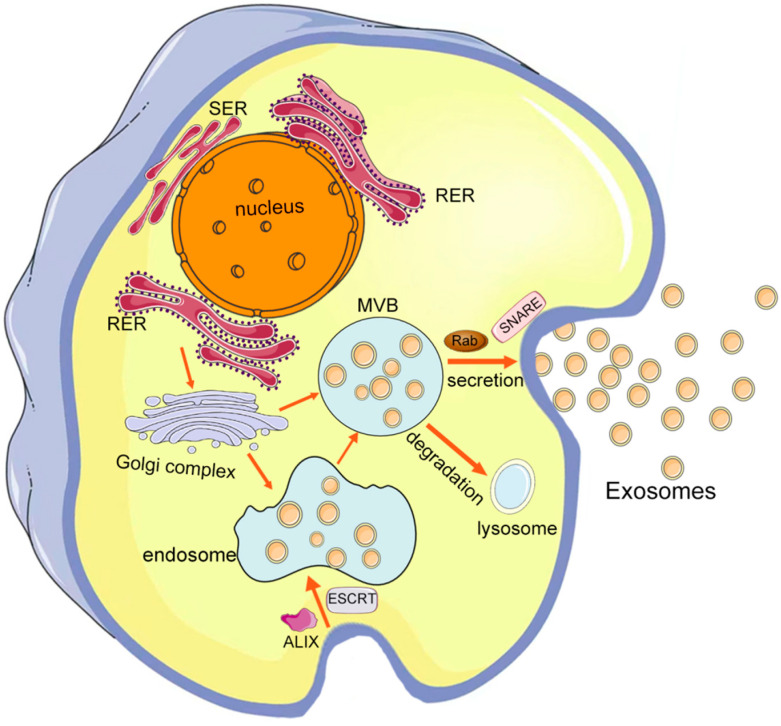
Biogenesis of exosomes. Cells produce small vesicles by endocytosis, which fuse to form endosomes, and are subsequently accompanied by the entry of nucleic acids, proteins, etc. into the endosomes. The endosomes gradually evolve into multivesicular bodies (MVBs), which are subsequently released extracellularly to form exosomes. RER: rough endoplasmic reticulum; SER: smooth endoplasmic reticulum; MVB: multivesicular body; ESCRT: endosomal sorting complex required for transport.

EVs are small membranous vesicles released into the extracellular matrix by almost all cells and are widely present in various body fluids [14]. It was initially thought that EVs were cell-secreted garbage bags as a cellular self-cleaning mechanism. Nevertheless, as research progressed, EVs were found to stably carry important signaling molecules such as proteins, mRNAs, miRNAs, and lipids, which have important roles in intercellular communication [15,16], disease diagnosis [17,18,19], disease development [20,21], and disease therapy [22,23]. EVs are a highly heterogeneous group of vesicles, which are broadly classified into three categories according to their biogenesis, size, and biophysical property: exosomes, microvesicles, and apoptotic bodies [14,24,25,26]. Among them, exosomes have received the most attention in recent decades. Exosomes are approximately 40–100 nm in diameter and float at a density of 1.13–1.19 g∙mL^−1^ in sucrose density gradient solutions [27,28,29,30]. The biogenesis process of exosomes is shown in Figure 1. It is worth noting that, according to the MISEV 2018 guidelines, it is difficult to fully purify and distinguish the above EVs due to methodological difficulties in isolation, and the term “exosomes”, even though widely used, is suggested to be replaced by the term “small extracellular vesicles (sEVs)” instead [31].

**Figure 2:**
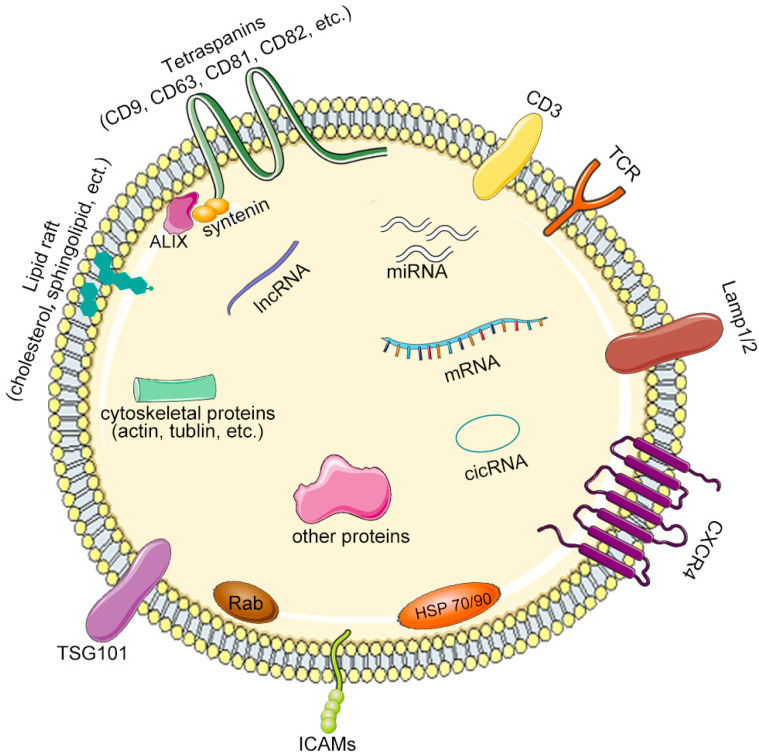
Rich contents and surface markers of exosomes. Exosomes are rich in cholesterol and sphingomyelin and have a lipid bilayer structure. Universally, exosomes contain tubulin, heat shock proteins, actin-binding proteins, ALIX, tetra membrane proteins, abundant nucleic acids, etc. CXCR4: CXC chemokine receptor 4; TCR: T cell receptor; HSP: heat shock proteins; ICAMs: intercellular cell adhesion molecules; TSG: tumor suppressor gene; lncRNA: Long non-coding RNA; miRNA: micro-RNA; cicRNA: circular RNA.

Considering the important function that exosomes contain diverse active molecules (Figure 2) and are capable of mediating cellular communications, they were vigorously developed as DDSs for the treatment of various diseases [32]. While liposomes have been used maturely in DDSs, we still cannot ignore their limitations as well as other synthetic nanoparticles, such as toxicity, immunogenicity, and short half-life due to rapid clearance by the mononuclear phagocytic system (MPS) [33]. Although PEG modification can improve the above defects to a certain extent, it may cause the acceleration of the blood clearance (ABC) phenomenon after repeated multiple dosing [34,35,36]. In addition, studies have shown that exposure to chemicals and cosmetics in daily life could increase PEG antibodies in the human body [37]. In contrast, when exosomes were used in DDSs, they had better biocompatibility and lower immunogenicity due to characteristics such as an endogenous biological origin [38], and longer circulation times [39]. Moreover, exosomes from different sources had different targeting capabilities and roles in crossing biological barriers [40,41].

Since the focus of this review was the study on exosomes as CNS drug delivery tools, all authors reached a consensus on article screening. More specifically, the review was performed in PubMed and Web of Science databases using the keywords (exosomes/extracellular vesicles/CNS/brain/spinal cord/drug delivery/therapeutic molecules). At the same time, articles of potential interest should include at least three of the above keywords in their title or abstract.

## 2. The Roles of Exosomes in the CNS

The dense BBB protects the brain from various pathogenic microorganisms but is also the biggest obstacle to treating CNS disease. Thus, designing a safe, effective, and specific DDS to cross the BBB is the focus of current research. Numerous drug delivery technologies have been devoted to crossing the BBB [42,43,44], among which exosomes exhibited more unique roles and advantages.

### 2.1. The Blood-Brain Barrier and Its Challenges in the Treatment

The BBB consists of brain capillary endothelial cells (BCECs), the basement membrane of pericytes, and astrocyte end-feet [45,46,47]. In addition, the presence of enzymes and efflux pumps (e.g., P-glycoproteins and multi-drug resistance proteins (MRPs)) restricts non-specific translocation [48]. The current approaches to facilitate the passage of therapeutic compounds across the BBB can be summarized in the following three methods: (i) invasive methods; (ii) pharmacological methods; (iii) physiological methods [49]. Intracerebral or intrathecal injection directly opens the blood-brain barrier, which is highly invasive and makes it easy to cause tissue trauma or inflammation. The pharmacological approach emphasizes drug modification. However, it is not sufficient and requires the assistance of delivery tools to maximize the drug’s effect. In the physiological approach, various nano-delivery systems including exosomes are used to cross the BBB.

### 2.2. Multiple Functions of CNS Cell-Derived Exosomes

Microglia, astrocytes, oligodendrocytes, and neurons can actively secrete exosomes [50,51,52,53] and play important roles in intercellular communications [54]. In the CNS, exosomes propagate signals not only over short distances within cells but also throughout the brain via the cerebrospinal fluid (CSF) [55]. Therefore, exosomes are involved in the maintenance of the normal physiological state of the CNS as well as in the development of pathology [56,57].

As the most fundamental structural and functional unit of the CNS, neurons play important roles in maintaining neural homeostasis. It was found that exosomes have astonishing efficacy in neurogenesis and repair [58]. Additionally, they can mediate intra-neural communication and make it better [59]. The delivery of miR-132 to vascular endothelial cells by neuronal exosomes could maintain cerebral vasculature integrity [60]. Similarly, neuronal-derived extracellular vesicles could also detect insulin/mTOR pathway changes in the brain of Down syndrome (DS) patients [61]. 

Microglia are important neural immune cells with active immune sensing functions [62]. Furthermore, exosomes secreted by microglia are actively involved in physiological and pathological processes in the CNS. For example, after traumatic brain injury, upregulation of miR-124-3p in microglia exosomes suppressed neuronal inflammation and promoted nerve growth via transfer to neurons [63]. Microglia could sense abnormal signals and convert from the inflammation-suppressing M2 type to the pro-inflammatory M1 type in pathologic conditions such as trauma and inflammation. Nevertheless, the involvement of exosomes may reverse this process, as exosomes from mesenchymal stem cells attenuated CNS inflammation and demyelination in EAE rats by regulating microglia polarization [64].

One important mediator of cell-to-cell communication is miRNA. For example, exosomes could transfer miR-133b from pluripotent mesenchymal stromal cells (MSC) to neuronal cells to promote nerve growth [65]. MSC-origin exosomes contained miRNAs that regulate neuroinflammation [66]. Exosomes secreted from glia and neurons mediated Alzheimer’s disease (AD) development via miRNA delivery. Changes in exosomal miRNAs in the peripheral blood or cerebrospinal fluid of patients were important guides for the early diagnosis of AD. More information on the roles of exosomal miRNAs in the diagnosis and treatment of AD have been reported [67].

Astrocytes are involved in the BBB formation, which may help regulate the ionic microenvironment surrounding neurons. Nevertheless, it has been shown that increasing the release of their exosomes can mitigate β-amyloid-induced neurotoxicity [68], offering novel ideas for the treatment of AD. However, the release of astrocyte exosomes is not always beneficial, and it has been shown that the microenvironment induced by astrocyte exosomal microRNA induced PTEN loss and brain metastatic growth of tumors [69].

Neural stem cells, in contrast to the aforementioned CNS cells, have the capacity for self-renewal and can differentiate into neurons, astrocytes, oligodendrocytes, etc. Neural stem cell exosomes held great promise in the treatment of neurodegenerative diseases [70], e.g., human neural stem cell exosomes have a protective effect against Parkinson’s disease [71] and could ameliorate AD [72]. Alongside this, extracellular vesicles derived from human neural stem cells have been shown to have good efficacy in the functional recovery of strokes in mice [73].

## 3. Strategies for Drug Loading into Exosomes

Although naked exosomes have therapeutic effects, they are limited. Drug-loaded exosomes can synergize the effects of drugs and exosomes, which have become a hotspot in DDS research.

The drug delivery system consists of two main components: one is the “outer shell”, as well as the drug delivery vehicle, which in this review refers to as exosomes. The other part is the “content”, which is also called the therapeutic drug. Currently, almost 20% of exosome-related topics are focused on drug loading, demonstrating its importance.

In contrast to the nano-formulations already developed, exosomes are loaded with active molecules more flexibly and diversely. Synthetic drug delivery vehicles like nanomaterials can be loaded with drugs during the synthesis. Exosomes, on the other hand, seem to be more receptive to the idea of loading active molecules before or after their secretion.

### 3.1. Pre-Secretory Loading 

The idea of loading drugs before exosome secretion, i.e., at the biogenesis stage of exosomes, is to manipulate exosome-generating progenitor cells, which is also called cell engineering. The donor cells are capable of secreting exosomes containing the drugs.

Drugs can enter cells by transfection, e.g., transfecting HEK293T cells with circSCMH1 plasmid [74] can efficiently encapsulate them into secreted exosomes, and targeted delivery to the brain can enhance neuronal plasticity and inhibit glial reactivity, thus effectively ameliorating stroke symptoms in mice and rhesus monkeys. Ge et al. [75] transfected miR-124-3p mimics into microglia for 6 h, and exosomes containing miR-124-3p were extracted and isolated 48 h after the culture medium was changed. Intravenous injection of the above exosomes alleviated the neurodegenerative lesions in repetitive mild traumatic brain injury (rmTBI) mice.

Drugs can also be co-incubated with donor cells to gain access to the cell interior. For instance, macrophage RAW264.7 cells co-cultured with high doses of curcumin at 37 °C for 24 h were isolated by ultracentrifugation to produce exosomes containing curcumin and were effective in alleviating symptoms of middle cerebral artery occlusion (MCAO) in vivo [76].

The method of drug loading by manipulating donor cells before exosome secretion is simple to operate. However, this strategy has high requirements for the compatibility between parental cells and a drug [11], and a variety of uncontrolled factors such as cell status and culture conditions ultimately lead to unstable or low loading efficiency [77,78]. In conclusion, a lack of knowledge of the complex intracellular metabolic pathways is the main reason. A better understanding of the mechanisms by which therapeutic molecules are transported in cells and localized to exosomes may improve loading efficiency.

### 3.2. Post-Secretory Loading

**Figure 3:**
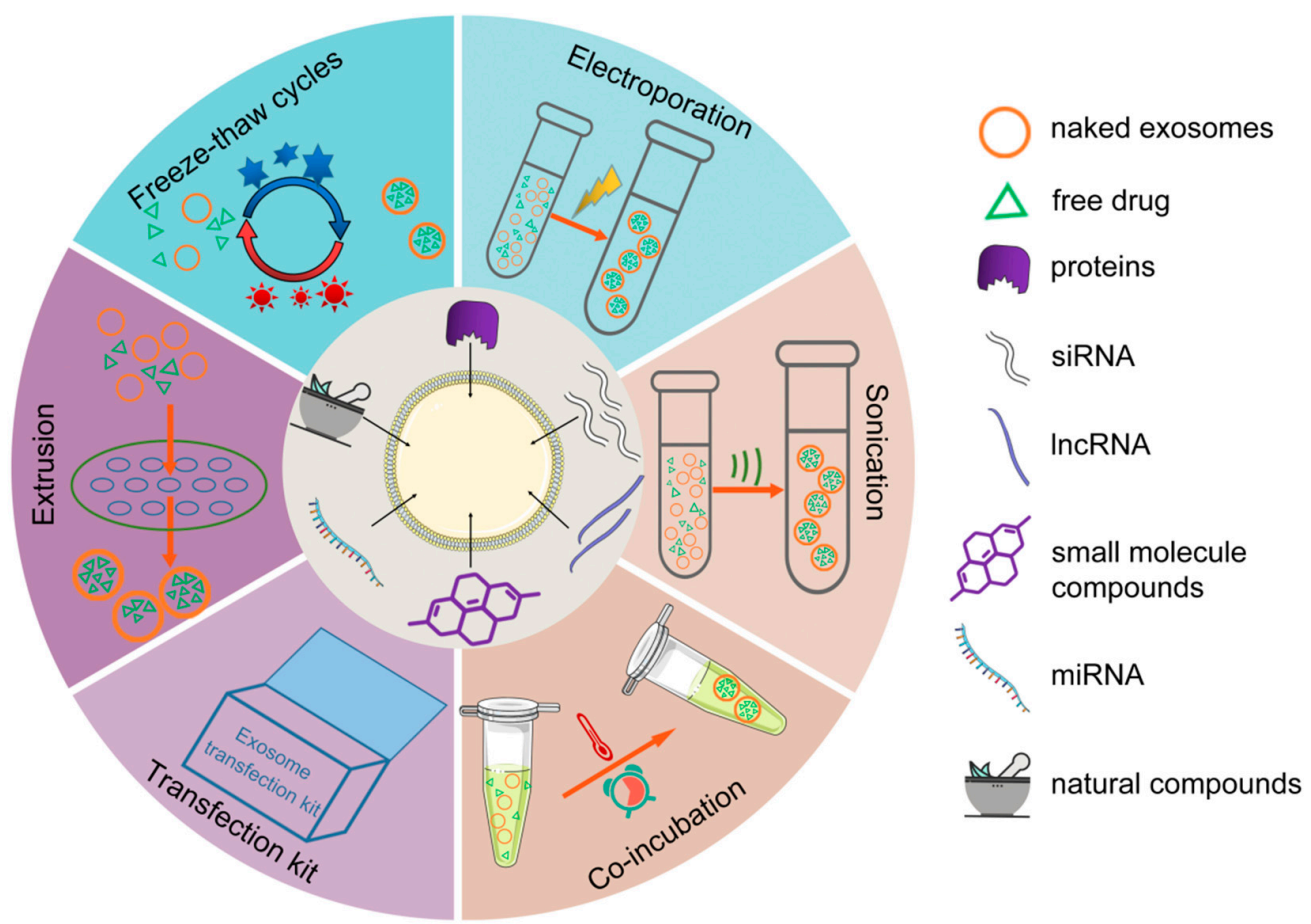
Post-secretory loading methods of exosomes. Drug encapsulation strategies for isolated exosomes include sonication, electroporation, co-incubation, freeze-thaw cycles, extrusion, transfection kits, etc.

The post-secretory approach refers to the way that naive exosomes are extracted and isolated from donor cells, and then the purified exosomes are loaded with drugs by various methods in vitro. Some common approaches include co-incubation, electroporation, sonication, repeated freeze-thawing, extrusion, and so on (Figure 3).

Exosomes have lipid bilayers, so co-incubation of exosomes with hydrophobic compounds allows passive entry of drugs into exosomes. For example, aptamer F5R1 was co-incubated with exosomes derived from the human embryonic kidney (HEK) 293T cells at 37 °C for some time [79], and an aptamer was able to efficiently enter exosomes and reduce a-synuclein aggregation, exerting a therapeutic effect on PD. It is worth mentioning that drug saturation solutions can be used to improve the efficiency of drug entry in vitro. This idea was applied by Qu et al. [80] to the experiment of dopamine loading in mouse blood exosomes. The enormous concentration difference contributed to the passive penetration of dopamine into exosomes during co-incubation. Dopamine-loaded exosomes showed a PD therapeutic effect. Co-incubation loading efficiency is related to drug hydrophobicity, molecular weight, exosome size, the lipid content of exosomes, and so on [81]. Therefore, the appropriate mixing ratio must be mastered to achieve higher loading efficiency.

Electroporation enables the membranes of exosomes to temporarily create pores in the presence of an electric current, through which the drugs enter the exosomes. For example, Zhang et al. [82] loaded ZIKV-specific siRNA into exosomes derived from HEK 293T cells at 450 V and 100 mF. They could cross the BBB and placental barrier to treat fetal microcephaly caused by ZIKV infection. Electroporation charging efficiency can be affected by voltage, electrical capacitance, discharge time, and other conditions.

Sonication is also commonly used. Luo et al. [83] isolated exosomes from fresh bovine milk and encapsulated epicatechin gallate (ECG) into exosomes using ultrasonication with a loading efficiency of 25.96%. The ultrasonically loaded exosomes were anti-apoptotic and neuroprotective against Parkinson’s disease. The loading efficiency of sonication is high but may lead to the aggregation of exosomes.

Aside from the abovementioned modalities, chemical reactions have also been skillfully applied [84]. LJM-3064 potently inhibits the inflammatory response and reduces the areas of demyelinating lesions in CNS by chemically covalently binding to amine groups on exosome surfaces via EDC/NHS. There are also alternative drug loading methods using exosome transfection reagents, which can directly transfer nucleic acids including siRNAs, miRNAs, mRNAs, and even plasmid DNAs into isolated exosomes [85]. These novel ways provide new ideas that may expand the development of exosomal drug delivery.

The addition of some adjuvants like saponin will improve loading efficiency appropriately. For example, in loading catalase, the authors [86] investigated the drug loading efficiency with and without saponin separately when using co-incubation as a modality and demonstrated that the addition of saponin significantly increased the drug loading efficiency from 4.9% to 18.5%. It was worth mentioning that the study also explored comparing the differences in drug loading rates of four modalities loaded on the same drug, and it was found that the loading efficiencies of sonication and extrusion were higher, 26.1% and 22.2%, respectively. However, this may not apply to other research, and the optimal method still needs to be explored personally in the respective studies. In some cases, multiple methods may be used at the same time to improve the efficiency of drug loading. For example, to load curcumin into mouse embryonic stem cell (MESC)-derived exosomes, Anuradha et al. [87] first incubated curcumin with exosomes in a 1:4 ratio for 15 min at room temperature, followed by rapid freeze-thawing two to three times. Successfully loaded exosomes were capable of reducing vascular inflammation and promoting neurovascular recovery in mice with ischemia-reperfusion injury.

In conclusion, efficient drug loading remains one of the biggest challenges. Especially for small molecules or extremely hydrophobic molecules, although simple co-incubation is the most commonly used method, it no longer meets the requirements. Therefore, Haney et al. [88] explored the optimization of co-incubation conditions. The team found that when the pH of the co-incubation system was adjusted to 8.0, the drug loading rate increased significantly. The possible reason was that a pH close to pI reduced the molecule’s charge and increased its hydrophobicity. It is reasonable to assume that optimizing other incubation conditions such as appropriate temperature increase, shaking, longer incubation time, etc. may also improve the loading rate. In addition, other suitable methods can be directly used instead of co-incubation. For example, mild sonication could significantly increase the loading rates of small molecules such as doxorubicin [88], paclitaxel [88,89], and gemcitabine [90].

Drug loading after secretion is controlled and efficient. Disadvantages, however, include destruction of exosome integrity, facile aggregation, etc. The character of the drug and the properties of exosomes may all influence the loading efficiency [91]. Therefore, the choice of drug loading mode is ultimately based on experimental needs.

## 4. Drug Administration Affects the Efficiency of Exosomes into the CNS

In DDS, appropriate changes in administration modes may enhance the therapeutic effect. Exosomes biodistribution was determined by cellular origin, route of administration, and targeting [92,93]. Thus, intravenous [94,95,96], nasal [97,98,99,100], intraperitoneal [101,102,103], and local [104] injections have been used extensively.

When exosomes are used as drug delivery tools, the most common administration is intravenous injection. For example, brain endothelium-derived exosomes might reach the brain after intravenous injection to deliver doxorubicin for the treatment of brain cancer in zebrafish [105]. Nevertheless, intravenously injected exosomes have restricted CNS tropism. In addition to this, the half-life of intravenous exosomes is shorter. Therefore, most of them are still surface-modified. One of the most widely used is the modification of neuron-targeting peptides (RVG) to enhance their brain-targeting capacity. 

Intranasal injection allows drug delivery to the brain via intranasal deposits, olfactory bulbs, trigeminal nerves, and the respiratory epithelium [106,107]. It is therefore widely used in brain disorders [108]. For example, exosomes containing cholesterol-modified AMO181a were capable of brain delivery and exerted therapeutic effects on ischemic brain injury when administered intranasally [98]. MSC-exosomes injected intranasally could cross the BBB and migrate to the injured area of the spinal cord, achieving recovery [109].

The local administration has also attracted attention. In a recent study, Han et al. [104] explored the use of an array of patches using autonomously designed gel microneedles loaded with 3D cultured MSC-exosomes to achieve in situ repair of exosomes in spinal cord injury. MSC-exosomes could reduce the neuroinflammatory response, promote polarization of microglia to M2 phenotype, and reduce glial scar formation to facilitate the repair of neurological injury. This successful exploration will also bring more researchers to think, innovate, and achieve breakthroughs concerning the exosomes administrations.

## 5. Engineering Exosomes Enhance the CNS Targeting and Therapeutic Efficacy

Exosomes inherit the characteristics of donor cells [110] and have certain passive targeting abilities, also known as a homing ability [111,112]. However, this natural homing ability is weak and may be affected by various factors such as injection methods, drug doses, and individual differences [92], thereby weakening or even losing the CNS targeting capacity of exosomes. Moreover, it has been demonstrated that intravenously injected naked exosomes were easily enriched in reticuloendothelial system (RES) organs such as the liver and it was extremely difficult for them to reach the CNS [92,93]. 

Engineered exosomes are endowed with desired targeting capabilities because they are decorated with various functional molecules on their surfaces [113,114,115,116]. Functional molecules can utilize specific ligand/receptor binding strategies to increase brain targeting capabilities [110,117]. Strategies for surface modification of engineered exosomes include genetic engineering and chemical modification [117,118].

Genetic engineering modification of exosomes is the fusion of the gene sequence of a target functional protein or peptide with a selected exosomal membrane protein, and then donor cells transfected with the above plasmids secrete engineered exosomes with targeting ligands on their surface [119]. Lamp2b-RVG is the most widely used hybrid plasmid for genetically modified brain-targeted exosome modifications. Lamp2b is a highly abundant membrane-localized protein on exosomes. Rabies virus glycoprotein (RVG) is the more recognized brain-targeting peptide, and RVG selectively targets neurons and brain endothelial cells by binding to the nicotinic acetylcholine receptor (nAChR) [120]. For example, Alvarez-Erviti et al. [118] genetically engineered Lamp2b-RVG plasmids and transfected autologous-derived dendritic cells to make them secrete engineered exosomes. Intravenous injection of the above exosomes specifically delivered siRNA to neurons, microglia, and oligodendrocytes in the brain, leading to specific gene knockdown and offering new hope for AD treatment. Genetic engineering strategies are currently widely used, but the procedures are complex and costly.

The chemical modification enables in vitro manipulation after exosome isolation. For example, stearoyl-RVG was inserted into the phospholipid bilayer of immature DC exosomes via hydrophobic interactions. The drug-loaded exosomes were able to scavenge α-synuclein aggregates and reduce cytotoxicity in PD neurons [121]. Han et al. [122] synthesized RVG-15-PEG-DSPE and inserted it onto the surface of exosomes containing doxorubicin to achieve the treatment of glioblastoma. In addition to RVG, other types of brain-targeting small molecules can also be engineered by chemical ligations. For example, to attach RGD peptides to the surface of exosomes, Tian et al. [123] first reacted naive exosomes isolated from MSC cells with DBCO-sulfo-NHS to produce EXO-DBCO, which was chemically reacted with the exposed amino groups on the surface of exosomes using NHS. Then, EXO-DBCO was reacted with the azide-modified cRGD in a click reaction, so the azide-modified cRGD was successfully attached to EXO-DBCO. Finally, the attachment of the targeted functional peptide cRGD to the surface of the exosomes by the chemical reaction was achieved (known as the bioorthogonal reaction) [124,125,126,127]. Jia et al. [128] also modified cargo-loaded exosomes with the glioma-targeting peptide RGE by click chemistry and finally obtained engineered exosomes with therapeutic effects on glioma. The chemical modification strategy is less costly and has higher linkage efficiency, but has more influencing factors such as temperature, solvent, etc.

Although current engineered exosomes focused on increasing targeting, Kojima et al. [129] pioneered the idea of increasing exosome release. Specifically, Kojima’s team constructed a triple hybrid plasmid with STEAP3 capable of participating in exosomes biogenesis, and syndecan-4 (SDC4) capable of supporting endosomal membrane outgrowth to form polycystic and L-aspartate oxidase (NadB) capable of promoting cellular metabolism by regulating the citric acid cycle. The combined expression of these genes significantly increased exosome production.

The aim of engineered exosomes is to overcome some deficiencies of naked exosomes and enable them to better serve as CNS drug delivery tools.

## 6. Research and Applications of Exosomal Drug Delivery Systems in CNS Diseases

As exosomes have been studied more intensively, numerous studies have been conducted on various CNS disease models to assess the efficacy of exosomal drug delivery systems and help their clinical translation.

### 6.1. Alzheimer’s Disease

Alzheimer’s disease (AD) is a progressive neurodegenerative disorder manifested by memory loss and cognitive decline [130]. There are no available therapeutic agents and the current drugs merely alleviate the associated symptoms. Neurogenic fibrillary tangles (NFT) composed of amyloid β (Aβ) plaques and abnormally phosphorylated tau protein (p-tau) are the major pathological hallmarks and AD is accompanied by neuronal death, synaptic loss, neuroinflammation, and brain injury [131,132,133].

Exosomes contain a wide variety of proteins, RNAs, etc., and therefore can mediate intercellular communication [134]. Because of this, exosomes have excellent performance in delivering RNA [135,136,137]. Small interfering RNAs (siRNAs) are among the many RNA drugs widely used, which result in gene silencing [138]. In recent years, siRNAs delivery technology has advanced rapidly, but extrahepatic and especially brain siRNA-targeted delivery has still been in the exploratory stage. Alvarez-Erviti et al. [118] made the first attempt to achieve intracerebral targeted delivery using exosomes as siRNA delivery vehicles. This study used self-derived dendritic cell exosomes to minimize immunogenicity, as well as RVG neuro-targeting peptides to modify exosomes. Suitable siRNAs were designed for the AD therapeutic target BACE1, resulting in a strong knockdown of the corresponding mRNA and proteins in mice.

### 6.2. Parkinson’s Disease

PD is the second most common neurodegenerative disorder worldwide, the major pathological hallmark being dopaminergic (DA) neuron degenerative death [139,140,141].

Catalase has been shown to have a protective effect on neurons. With this in mind, Haney et al. [86] explored the use of different methods for loading catalase into RAW264.7 cell exosomes. After intranasal administration, catalase-loaded exosomes were taken up by neurons and microglia of PD mice and exhibited significant neuroprotective effects. Likewise, Kojima et al. [129] used a mixed plasmid containing peroxidase mRNA along with RVG to transfect HEK-293T cells. By subcutaneous injection of the cells, the cells produced exosomes containing peroxidase mRNA in vivo and delivered it to the brain and alleviated the neurotoxicity and neuroinflammation caused by PD.

Alpha-synuclein aggregation is closely related to Lewy body formation and dopaminergic neuron death and plays an important role in the pathogenesis of PD. Thus, the reduction of α-synuclein protofibril aggregation is an idea for PD therapy. Cooper and colleagues [142] showed a significant reduction in α-Syn mRNA and protein levels in the brains of both normal and PD mice treated with RVG exosomes loaded with anti-α-Syn siRNA. This team targeted the reduction of α-synuclein aggregation again [143] using primary dendritic cell-derived, RVG-modified exosomes encapsulating the therapeutic molecule shRNA-MCs, which were specifically delivered to the brains of PD mice by intravenous injection and attenuated α-synuclein aggregation, reducing the loss of dopaminergic neurons and thus improving PD-related symptoms. It is noteworthy that the authors considered that shRNA size could affect drug delivery efficiency during exosome electroporation and specifically designed a smaller loop shape, i.e., shRNA-MCs, to achieve better loading efficiencies and therapeutic effects. This novel approach provided insights into the exosomal drug delivery systems for the treatment of other neurodegenerative disorders. Along similar lines, RVG-exosomes loaded with aptamer F5R1 were also able to deliver to neurons in vitro and in vivo, significantly reducing the pathological aggregations induced by α-synuclein pre-formed fibers (PFFs) [79].

Dopamine is also used for PD in addition to the treatment options mentioned above, but dopamine reaching the brain is problematic. Qu et al. [80] used blood exosomes to encapsulate dopamine and successfully delivered it to the striatum and substantia nigra of the brains by intravenous injection to ameliorate symptoms of PD mice.

### 6.3. Huntington’s Disease

Huntington’s disease (HD) is a hereditary neurodegenerative disorder [144]. Disease-causing genes, also known as Huntington’s genes, produce abnormal Huntington’s proteins which tend to adhere and aggregate, ultimately resulting in neuronal cell death [145,146]. Thus, silencing this gene is a strategy for HD therapy. Since exosomes have been widely used for siRNA delivery [147], Didiot et al. [148] modified siRNA hydrophobically to improve loading efficiency when incubated with exosomes. After unilateral infusion of exosomes into the mice striatum, exosomes carried siRNA for diffusion to the contralateral striatum and transported it to neurons, ultimately resulting in dose-dependent silencing of Huntingtin mRNA and protein in bilateral ventricles.

### 6.4. Stroke

A stroke is a general term for brain tissue damage caused by sudden rupture or blockage of cerebral blood vessels, including ischemic and hemorrhagic stroke [149,150,151]. It is a neurological disease with a high rate of disability worldwide [152,153].

Subarachnoid hemorrhage is a major reason for hemorrhagic stroke. In one study [154], miR-193b-3p was delivered to the brains of mice by intravenous injection of exosomes, which ameliorated inflammatory response through inhibition of HDAC3. Lastly, neurobehavioral disturbances, brain edema, BBB injury, and neurodegeneration were alleviated.

Compared with hemorrhagic stroke, ischemic stroke accounts for the vast majority of the total number of strokes [155] and the main causes are occlusion and stenosis of the associated arteries. MiRNAs were involved in regulating nervous system development and function [156,157] and their therapeutic potential has been demonstrated in a variety of CNS diseases [158,159]. MSC-exosomes showed superior effects in the treatment of various neurological disorders [160,161,162], as evidenced by neural repair and inhibition of neuroinflammation [95,163]. Therefore, several studies [164,165,166] have explored the use of MSC-exosome-encapsulated miRNAs as therapeutic molecules for the treatment of ischemic strokes. For example, Zhao et al. [164] transfected MSCs with miR-223-3p to generate miRNA-loaded exosomes. After intravenous injection, the volume of cerebral infarction was greatly reduced, and neurological function was markedly improved. Its protective effect was closely associated with an increased polarization of microglia towards the M2 phenotype. MiR-124 also biased neural progenitor cells toward neuronal lineage through exosomes, greatly contributing to neurogenesis after ischemia [165]. Similarly, miR-17-92-enriched exosomes improved neurological function and promoted neurogenesis [166].

Curcumin is a natural polyphenolic compound with a potent anti-inflammatory effectivity [167,168]. RGD peptides were bound to the surface of MSC-exosomes by bioorthogonal chemistry, and curcumin entered the exosomes after co-incubation. Drug-loaded exosomes targeted microglia, neurons, and astrocytes in the brain following intravenous injection and effectively inhibited inflammatory response and apoptosis in ischemic brain injury areas [123]. Whereas the aforementioned brain targeting of exosomes was RGD mediated, He et al. [76] took advantage of the targeted migration capability of mouse macrophage RAW264.7 exosomes themselves, loading curcumin as a therapeutic agent as well. After intravenous injection, the exosomes targeted ischemic areas and exerted powerful neuroprotective effects by eliminating ROS generation and mitigating BBB destruction. Exosomes from mouse embryonic stem cells (MESC) may also bind curcumin for stroke treatment [87].

Aside from the curcumin and miRNAs mentioned above, other active molecules such as circSCMH1 [74], recombinant human NGF mRNA [169], and pigment epithelium-derived factor (PEDF) [170] could also be coated with exosomes as therapeutic molecules for strokes treatment.

### 6.5. Brain or Spinal Cord Injury and Neuroinflammation

Brain or spinal cord injury is a neuroinflammatory condition that endangers human health. In spinal cord injury (SCI), MSC-exosomes acquired the homing property of its progenitor cells and were able to automatically penetrate the BBB to reach the injured spinal cord region. Taking advantage of this property, Guo et al. [109] repaired SCI after intranasal injection of MSC-exosomes containing phosphatase and tensin homolog siRNA (PTEN-siRNA), which effectively reduced neuroinflammation and glial proliferation. In addition to SCI, repetitive mild TBI (rmTBI) not only triggered neuroinflammation but may also have led to long-term neurodegenerative diseases like AD [171]. Microglia, as specific resident macrophages in the CNS, play key roles in the development and regression of neuroinflammation [172,173]. One study [75] reported that intravenous administration of microglia exosomes harboring miR-124-3p could target neurons and alleviate neurodegeneration. Multiple sclerosis (MS) is a classic neuroinflammatory disorder mainly characterized by inflammatory demyelinating lesions in the white matter of the CNS [174]. The LJM-3064 aptamer, which was chemically covalently bound to the surface of MSCs exosomes, significantly ameliorated neuroinflammation by reducing areas of demyelinating lesions in the spinal cord and brain [84]. Zhuang et al. [175] verified the therapeutic effect of the exosomes’ drug delivery system in three neuroinflammatory models: LPS-induced encephalitis models, experimental autoimmune encephalomyelitis (EAE) models, and brain-tumor-induced neuroinflammation. Exosomes loaded with curcumin or JSI124 (a signal transducer and activator of the transcription 3 inhibitor) could target microglia and induce microglia apoptosis to alleviate neuroinflammation after intranasal injection.

### 6.6. Brain Tumor

Gliomas account for 40% to 50% of brain tumors and are the most common primary intracranial tumors. Glioma has a high mortality, disability, and very poor prognosis [176]. A major reason why current drugs have difficulty in achieving the desired therapeutic effect is the difficulty in crossing the BBB to accumulate sufficient local drug concentrations at the tumor site. Exosomes, as drug delivery systems capable of crossing the blood-brain barrier, have played important roles in glioma treatment. For example, a recent study developed exosomes modified with Angiopep-2 and transcriptional peptide trans-activators and loaded doxorubicin to reach gliomas by crossing the BBB, not only improving the survival time of glioma mice by more than 2-fold but also greatly reducing the toxic effects of doxorubicin [177]. Similarly, Jia et al. [128] loaded curcumin and SPIONs (MRI contrast agents) into exosomes simultaneously by electroporation and then modified the exosomes with glioma-targeting peptides by click chemistry, ultimately showing dual antitumor effects.

In tumor therapy, overcoming drug resistance is also a major task. Glioblastoma multiforme (GBM) often exhibit chemo- and radio-resistance [178]. Yamada’s group found that the main reason for the resistance of GBM to the chemotherapeutic drug temozolomide was the upregulation of miR-9 in cells, which was involved in the expression of the P-glycoprotein, a drug efflux transporter. Given this, the authors used mesenchymal stem-cell-derived exosomes carrying anti-miR-9 to eventually reverse the expression of multidrug transporter proteins and sensitize GBM cells to temozolomide [179].

Apart from gliomas, brain tumors contain astrocytoma, meningioma, neurofibroma, and so on [180]. Because of the complex and precise structures in the brain, whatever type of cytopathic brain tumor, once compressed to any part of the brain, will cause damage to different functions in the body. For the treatment of brain tumors, exosome delivery systems continue to perform well. For example, brain endothelium-derived exosomes could deliver the anticancer drug doxorubicin via the BBB for the treatment of brain cancer in zebrafish [105].

### 6.7. Other Brain Diseases: Viral Infection; Drug Addiction

Drug addiction is highly damaging to individuals and society, mainly because of a series of withdrawal symptoms that occur when the drug stops stimulating the CNS. Nevertheless, there are very limited treatments available [181]. Among them, opioids mediate analgesic and addictive effects through MOR receptors. Thus, Liu’s team boldly attempted to use exosomes derived from RVG-modified HEK 293T cells to deliver MOR siRNA specifically to the mouse brain and downregulate MOR expression to mediate morphine addiction treatment [182].

Viruses can invade the organism and proliferate in the host cells through multiple pathways. Even some viruses invade the CNS, such as the ZIKA virus, which could cross the placenta and BBB to enter the fetal brain and cause fetal microcephaly [82]. Zhang et al. [183] designed an RVG-modified exosome derived from HEK 293T cells, which contained ZIKV-specific siRNA. Once intravenously injected, exosomes could cross the mice’s placental barrier from the maternal circulatory system to the fetal mice and cross the fetal BBB to target the head, thus protecting the fetal mice from ZIKV virus infection and alleviating neuroinflammation.

In summary, exosomes have been widely used as CNS drug delivery tools. They can be loaded with a variety of therapeutic molecules, including nucleic acids, proteins, and small molecule compounds. Exosomal drug delivery systems have shown good results in treating almost all CNS diseases, from neurodegenerative diseases to neuroinflammatory diseases, from brain tumors to brain viral infections. This greatly encouraged researchers to continue to explore and utilize exosomes as drug delivery tools for the treatment of CNS diseases. Additionally, the studies on exosomal drug delivery systems for CNS diseases are summarized in Table 1.

## 7. Conclusions and Perspectives

According to the latest database (http://www.exocarta.org/, accessed on 23 September 2022), 9769 proteins, 3408 mRNAs, and 2838 microRNAs have been identified in exosomes. These rich contents make the role of exosomes in intercellular communication, disease development, and therapy increasingly expansive. It brings light to exosomes as CNS drug delivery tools. To date, exosome studies have involved a number of species including mice, rats, zebrafishes, and monkeys (https://evtrack.org/, accessed on 23 September 2022), which will drive the clinical translation of exosomes.

As CNS drug delivery tools, exosomes have several characteristics compared to synthetic nanoparticles: low immunogenicity, non-toxicity, high cargo carrying, protective capabilities [184,185,186], and the ability to cross the blood-brain barrier [187,188,189]. To improve the efficiency of entering the CNS, exosome surface engineering modification has been widely used. It can increase the local concentration of drugs at the lesion site and reduce toxic side effects while improving therapeutic efficacy. However, the structural and functional stability of engineered exosomes and the mechanism of improving CNS targeting efficiency have not been thoroughly studied. Therefore, more research is needed to enhance the stability, security, and standardization of these strategies.

There are various types of active molecules encapsulated in exosomes, including nucleic acids (miRNAs, siRNAs, shRNAs, mRNAs, etc.), small molecule compounds (doxorubicin, dopamine, etc.), natural products (curcumin, paclitaxel, resveratrol, etc.), and proteins (catalase, pigment epithelium-derived factor, etc.). For different types of therapeutic drugs and exosomes, various loading methods exist, both before and after the secretion of exosomes. Genetic engineering, co-incubation, electroporation, sonication, extrusion, and repeated freeze-thawing have been employed. On this basis, we need to further investigate the mechanisms of exosome internalization to better understand how they work and thus select suitable drug loading methods for different types of therapeutic molecules. There has been increasing evidence that exosomes carrying the therapeutic molecules mentioned above have made breakthroughs in various CNS diseases such as brain tumors, neurodegenerative diseases, multiple scleroses, brain or spinal cord injuries, strokes, drug addictions, and viral infections. However, progress in the isolation and purification of exosomes as well as mechanistic studies are needed to fully realize the potential of exosomes in CNS drug delivery systems.

**Figure 4:**
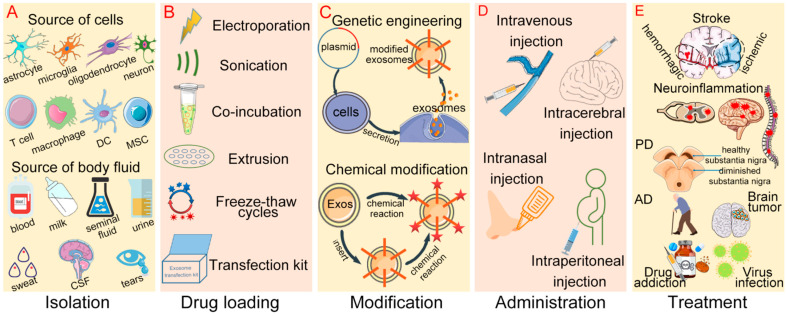
The research process of exosomes as CNS drug delivery tools. (**A**) Exosomes are isolated from cells or body fluids; (**B**) multiple drug loading modes of exosomes; (**C**) exosomes can be modified by genetic engineering and chemical reactions; (**D**) several administration routes of cargo-loading exosomes; (**E**) exosomes loaded with therapeutic molecules are used to treat a variety of CNS diseases. DC: dendritic cell; MSC: mesenchymal stem cell; CSF: cerebrospinal fluid; Exos: exosomes; PD: Parkinson’s disease; AD: Alzheimer’s disease.

In conclusion, engineering modifications, administration routes, type of therapeutic molecules, and encapsulation modes are all important parameters of exosomes as CNS drug delivery tools (Figure 4) and affect their clinical transformation. The majority of patients with CNS diseases will benefit if we can pay more attention to the problems already present, deepen our understanding of unexplained mechanisms, and realize their maximal clinical potential.

## Figures and Tables

**Table 1:** Preclinical studies on exosomes as DDS for CNS diseases treatment.

Disease	Donor Cell	Therapeutic Molecule	Drug Loading Method	Modification Strategy	Animal	Administration Route	Targeted Cells	Ref.
Alzheimer’s disease	self-derived dendritic cells	BACE1 siRNA	electroporation	Lamp2b-RVG	Mice	intravenous	neurons, microglia, and oligodendrocytes	[118]
Parkinson’s disease	HEK-293T cells	catalase mRNA	transfection	Lamp2b-RVG	Mice	subcutaneous	unknown	[129]
	primary dendritic cells	a-Syn siRNA	electroporation	Lamp2b-RVG	Mice	intravenous	unknown	[142]
	primary dendritic cells	shRNA-MCs	electroporation	Lamp2b-RVG	Mice	intravenous	unknown	[143]
	HEK-293T cells	aptamer F5R1	co-incubation	Lamp2b-RVG	Mice	intraperitoneal	microglia, neurons, and astrocytes	[79]
	mice blood	dopamine	co-incubation	None	Mice	intravenous	unknown	[80]
	RAW264.7	catalase	co-incubation, freeze-thaw, sonication, or extrusion	None	Mice	intranasal	neurons and microglia	[86]
Huntington’s disease	glioblastoma U87 cells	hsiRNA*^HTT^*	co-incubation	None	Mice	unilateral brain infusion	neurons	[148]
Stroke	mesenchymal stromal cells (MSC)	curcumin	co-incubation	c(RGDyK) peptide	Mice	intravenous	microglia, neurons, and astrocytes	[123]
	bone marrow mesenchymal stem cells (BMSC)	miR-193b-3p	electroporation	Lamp2b-RVG	Mice	intravenous	unknown	[154]
	HEK-293T cells	circ SCMH1	transfection	Lamp2b-RVG	Mice and rhesus monkeys	intravenous	microglia, neurons, and astrocytes	[74]
	mesenchymal stem cells (MSCs)	miR-223-3p	transfection	None	Rats	intravenous	unknown	[164]
	RAW264.7	curcumin	co-incubation	None	Rats	intravenous	neurons and endothelium cells	[76]
	mouse embryonic stem cells (MESCs)	curcumin	co-incubation	None	Mice	intranasal	astrocytes and neurons	[87]
	multipotent mesenchymal stromal cells (MSCs)	miR-17-92	transfection	None	Rats	intravenous	unknown	[166]
	adipose-derived stem cells (ADSCs)	PEDF	transfection	None	Rats	intravenous	unknown	[170]
	HEK-293T cells	recombinant human NGF mRNA	transfection	Lamp2b-RVG	Mice	intravenous	microglia, neurons, and astrocytes	[169]
	bone marrow mesenchymal stem cells (BMSC)	miR-124	electroporation	Lamp2b-RVG	Mice	intravenous	neurons, astrocytes, and oligodendrocytes	[165]
Repetitive mild traumatic brain injury (rmTBI)	microglia	miR-124-3p	transfection	None	Mice	intravenous	microglia, neurons, and astrocytes	[75]
Spinal cord injury (SCI)	mesenchymal stem cells (MSC)	PTEN-siRNA	co-incubation	None	Rats	intranasal	neurons	[109]
Multiple sclerosis	EL-4 cells	curcumin or JSI124	co-incubation	None	Mice	intranasal	microglia	[175]
	mesenchymal stem cells (MSCs)	LJM-3064 aptamer	EDC/NHS	None	Mice	intravenous	unknown	[84]
Brain tumor	brain endothelial cell (bEND.3)	doxorubicin	co-incubation	None	zebrafishes	intravenous	unknown	[105]
	RAW264.7	curcumin and SPIONs	electroporation	RGE-peptide	Mice	intravenous	glioma	[128]
ZIKV infection	HEK-293T cells	ZIKV-specific siRNA	electroporation	Lamp2b-RVG	Mice	intravenous	microglia, neurons, and astrocytes	[183]
Morphine addiction	HEK-293T cells	Mu (MOR) siRNA	transfection	Lamp2b-RVG	Mice	intravenous	neuro2A	[182]

## Data Availability

Not applicable.

## Abbreviations

CNS	central nervous system
RVG	rabies virus glycoprotein
DDS	drug delivery system
BBB	blood-brain barrier
AAV	adeno-associated virus
EVs	extracellular vesicles
MPS	mononuclear phagocytic system
PEG	polyethylene glycol
ABC	acceleration of blood clearance
CSF	cerebrospinal fluid
MSC	mesenchymal stem cell
SCI	spinal cord injury
TBI	traumatic brain injury
MS	multiple sclerosis
EAE	experimental allergic encephalomyelitis
RES	reticuloendothelial system
PD	Parkinson’s disease
AD	Alzheimer’s disease
